# EP19 Renal sarcoidosis associated with certolizumab pegol treatment for psoriatic arthritis: a case report

**DOI:** 10.1093/rap/rkaa052.018

**Published:** 2020-11-03

**Authors:** Ryan Malcolm Hum, Durga Kanigicherla, Pauline Ho

**Affiliations:** r1 The Kellgren Centre for Rheumatology, Manchester Royal Infirmary, Manchester, United Kingdom; r2 Manchester Institute of Nephrology & Transplantation, Manchester Royal Infirmary, Manchester, United Kingdom

## Abstract

**Case report - Introduction:**

Sarcoidosis is a systemic granulomatous disease without a known aetiology. Patients can be asymptomatic and do not require treatment. However, for the severe cases of sarcoidosis, anti-tumour necrosis factor-α (TNF-α) agents have been used in addition to corticosteroids and disease modifying anti-rheumatic drugs (DMARDs). Paradoxically, anti-TNF-α treatment has also been shown to induce sarcoidosis in patients with autoimmune diseases, though what is known about the pathogenesis of this phenomenon is limited. We present a case of renal sarcoidosis occurring following Certolizumab Pegol (CZP) treatment in a patient with psoriatic arthritis.

**Case report - Case description:**

A 55-year-old Caucasian man presented to the emergency department in Jan 2020 with a seizure and was found to have a stage 3 acute kidney injury.

He was diagnosed with psoriatic arthritis (PsA) at age 47 and plaque psoriasis at 20. He has no other past medical history. For PsA, he was initially treated with Methotrexate, but this was stopped due to nausea. After failing on sulfasalazine, he was started on Humira (Adalimumab) and low dose Methotrexate, 10mg weekly. Due to lack of efficacy, he switched to Benepali (Etanercept) monotherapy. One year later, he was switched to Cimzia (Certolizumab Pegol) due to worsening disease activity. He was on the treatment for 7 months before presenting with seizures and renal failure.

His seizure was caused by uraemia and hypertension secondary to renal failure. He has severe renal dysfunction (eGFR of 8ml/min/1.73m2), but low-level proteinuria (uPCR of 47mg/mmol). A renal biopsy was performed which showed features of non-necrotising interstitial nephritis, with well-formed granulomas & multiple giant cells, consistent with renal sarcoidosis. Infections were excluded including TB.

He was started on high dose prednisolone 80mg daily, a modest improvement in creatinine was observed from 636µmol/L (eGFR 8ml/min/1.73m^2^) on admission to 526µmol/L (eGFR 10ml/min/1.73m^2^) on discharge a week later. He did not require renal replacement therapy during admission. The dose of prednisolone was tapered over time, he is now on 10mg per day and his creatinine in April 2020 was 344 µmol/L, (eGFR 16 ml/min/1.73m^2^).

Currently, his psoriatic arthritis is under control, and his latest creatinine in July was 293 µmol/L (eGFR 20ml/min/1.73m^2^), his prednisolone was reduced further to 5mg daily.

In this case report, we reviewed the evidence on anti-TNF-α induced sarcoidosis and options for future biologic agent therapy if there were to be deterioration of psoriatic arthritis or flare of psoriasis.

**Case report - Discussion:**

Certolizumab-induced sarcoidosis is still a rare, but emerging phenomenon, with an unclear pathogenesis. Of the 87 published case reports of anti-TNF-α agent-induced sarcoidosis, 85 were in patients receiving Etanercept, Adalimumab, and infliximab. In terms of Certolizumab-induced sarcoidosis, a PubMed search using the terms “sarcoidosis, and Certolizumab”, found only two previously reported cases.

In the first case, a 45-year-old Caucasian woman with HLA-B27-positive non-radiographic axial spondyloarthritis developed severe renal insufficiency and mediastinal lymphadenopathy following 6 months of Certolizumab therapy. Renal biopsy showed granulomatous interstitial nephritis consistent with renal sarcoidosis, and bronchial biopsies showed non-caseating granulomas. The patient was treated with prednisolone 60mg daily, which was weaned over 6 months along with the initiation of Methotrexate and Secukinumab. This combination led to a minor improvement in her axial symptoms and stabilisation of her renal disease.

In the second case, a 69-year-old woman with rheumatoid arthritis developed fatigue, appetite loss and low-grade fever after 6 years of Certolizumab Pegol therapy. She was found to have granulomatous uveitis, bilateral hilar and mediastinal lymphadenopathy, a subcutaneous nodule on her right knee, and micronodules in the upper lobes of both lungs. A video-assisted thoracoscopic biopsy showed noncaseous epithelioid cell granulomas consistent with sarcoidosis. Furthermore, a biopsy of the nodule on her knee showed noncaseous epithelioid cell granulomas. She received 20mg of prednisolone daily to advantageous effect, which was then tapered to 10mg daily without recurrence of sarcoidosis. She was then started on abatacept treatment while further tapering her prednisolone.

**Case report - Key learning points:**

In these two cases plus this current case, discontinuation of anti-TNF-α agents with systemic corticosteroid treatment resulted in the regression of sarcoidosis.

One proposed mechanism of Certolizumab-induced sarcoidosis involves neutralising antibodies, though data on the impact and frequency of anti-drug antibodies in Certolizumab therapy is still unclear. Even the frequency of anti-drug antibodies in Certolizumab therapy is still unclear, with reported percentages ranging from 3.3% to 25.3%. Anti-drug antibodies with Adalimumab is thought to be more common with reported frequency as high as 38.0%, with 21 case reports published about Adalimumab-induced sarcoidosis.

There are also case reports on Secukinumab (anti-IL17) associated with onset of sarcoidosis, this is one of the treatment options for psoriatic arthritis and psoriasis as per NICE guidance (TA445). Tofacitinib has been approved by NICE recently (TA543) for PsA, so far, there are no case reports associated with the onset of sarcoidosis, rather, it has been used to treat sarcoidosis successfully. Therefore, Tofacitinib seems to be a potential treatment option for this patient.

Key learning points therefore include:

1. Certolizumab-induced sarcoidosis is a rare but increasingly recognised phenomenon.
2. The role of anti-drug antibodies in the pathogenesis of anti-TNF-α agent-induced sarcoidosis remains unclear.
3. Systemic corticosteroid treatment seems to be an effective treatment for anti-TNF-α agent-induced sarcoidosis.
4. Tofacitinib has not been reported to cause drug-induced sarcoidosis and is a viable alternative treatment option.

